# Assessment of Left Atrial Structure and Function by Echocardiography in Atrial Fibrillation

**DOI:** 10.3390/diagnostics12081898

**Published:** 2022-08-05

**Authors:** Mengmeng Ji, Lin He, Lang Gao, Yixia Lin, Mingxing Xie, Yuman Li

**Affiliations:** 1 Department of Ultrasound Medicine, Union Hospital, Tongji Medical College, Huazhong University of Science and Technology, Wuhan 430022, China; 2 Clinical Research Center for Medical Imaging in Hubei Province, Wuhan 430022, China; 3 Hubei Province Key Laboratory of Molecular Imaging, Wuhan 430022, China; 4 Shenzhen Huazhong University of Science and Technology Research Institute, Shenzhen 518057, China; 5 Tongji Medical College and Wuhan National Laboratory for Optoelectronics, Huazhong University of Science and Technology, Wuhan 430022, China

**Keywords:** left atrium structure and function, atrial fibrillation, echocardiography, three-dimensional echocardiography, speckle tracking echocardiography

## Abstract

Atrial fibrillation (AF) is the most common arrhythmia with significant morbidity and mortality. Exacerbated by the aging population, the prevalence of AF is gradually increasing. Accurate evaluation of structure and function of left atrium (LA) has important prognostic significance in patients with AF. Echocardiography is the imaging technique of first choice to assess LA structure and function due to its better availability, accessibility and safety over cardiac computed tomography and cardiac magnetic resonance. Therefore, the aim of this review is to summarize the recent research progress of evaluating LA size by three-dimensional echocardiography and LA function by speckle tracking echocardiography (STE) in predicting the occurrence and recurrence of AF and determining the risk of stroke in AF. In addition, we summarized the role of traditional echocardiography in detecting AF patients that are at high risk of heart failure or cardiovascular death.

## 1. Introduction

Atrial fibrillation (AF), with significant morbidity and mortality, is the most common arrhythmia around the world [1,2,3]. It is also an epidemic, with an estimated 33 million patients worldwide [4,5]. The incidence of AF is expected to increase as the elderly population with chronic cardiovascular conditions increases [4,6,7,8,9,10,11]. In addition, AF is independently associated with stroke, heart failure (HF) and cardiovascular death, and consequently, it contributes to a significant public health burden [7,12,13,14]. The left atrium (LA) is seen as a critical indicator of adverse outcomes of cardiovascular diseases, especially in patients with AF [15]. LA enlargement and subsequent dysfunction are usually obvious in patients with AF [16,17], and indicate that LA cannot adapt to the onset of rapid atrial tachyarrhythmias or the status of pressure or volume overload. Such LA enlargement is usually considered LA remodeling [15], an important mechanism of AF and also the main pathophysiological basis of AF progression [18]. Prompt treatment can attenuate this pathophysiologic process to a degree, and subsequent reverse remodeling leads to a decrease in LA size and improvement in LA function [15]. Several studies have demonstrated that the accurate evaluation of LA structure and function plays a critical role in characterizing the population with or at high risk of AF, predicting the AF recurrence and assessing the risk for stroke and other cardiovascular diseases [8,19,20]. Nowadays, LA multimodality imaging including echocardiography, cardiac computed tomography (CCT) and cardiac magnetic resonance (CMR) can comprehensively assess the size and function of LA by a variety of parameters. However, the image quality of CCT can be deteriorated when heart rate exceeds 60 beats per minute or arrhythmia occurs. Other disadvantages of CCT are its lower temporal resolution, low dose of radiation exposure and contraindications in patients with renal insufficiency and contrast hypersensitivity [12,21,22,23]. CMR is considered the gold standard for assessing LA size and function [8,12], and it can provide accurate endocardial border definition [24,25,26]. In addition, the detailed anatomic definition of the pulmonary vasculature can be evaluated by CMR, which can benefit AF ablation [15]. However, it has several limitations, including a longer scan time, high expense, and the relative contraindication with metal implant or claustrophobia, which limit the wide clinical application of CMR [27].

The aim of this review is to summarize the recent research evaluating LA structure and function by real-time three-dimensional (3D) echocardiography (RT-3DE) and 3D speckle tracking echocardiography (3D-STE) in identifying patients with or at high risk of AF and predicting AF recurrence. In addition, because RT-3DE and 3D-STE have not been widely applied in clinical practice, we also summarize the application of LA strain by two-dimensional STE (2D-STE) in predicting the new onset of AF and assessing the risk of stroke, as well as the role of traditional echocardiography in predicting HF and cardiovascular death in AF. 

We used the keywords, “LA structure and function”, “LA strain”, “STE”, “AF”, and “stroke”, to search the literature on PubMed and Web of Science from inception to 2022, mainly selecting the literature published in the last five years.

## 2. LA Structure and Function in AF

### 2.1. LA Structural Assessment in AF

LA structural remodeling is a result of increased interstitial fibrosis, which results in cardiac structural alterations including LA dilatation [15,28,29]. LA enlargement and LA fibrosis are not only the hallmark initiating factors of AF, but are also the critical reason for AF maintenance and progression. AF can be evaluated by LA size and myocardial fibrosis, and echocardiography is usually considered the most extensive and established method of evaluating cardiac structure [12]. LA dilatation is a significant indicator for predicting the morbidity and mortality of cardiovascular diseases [30]. However, it is difficult to accurately quantify the size of LA because of its complex and asymmetric geometry, intricate fiber alignment, and influence of LA appendage and pulmonary vein. Though the antero-posterior diameter (APD) of LA is widely used in clinical practice due to its high repeatability, the APD of LA cannot precisely reflect the practical size of LA due to asymmetric remodeling during dilation [18,20]. Previous studies have demonstrated that LA volume (LAV) could reflect the changes of LA in all directions when LA is remodeled [31]. The maximum LA volume index (LAVi) remains the only officially recommended predictor that is independently associated with cardiovascular adverse events in the current guidelines [32]. The full name and calculations of LA structural parameters are depicted in Table 1.

LA fibrosis is the basis of the occurrence and maintenance of AF and is closely related to the recurrence of AF after ablation [33]. Additionally, LA fibrosis is a critical factor associated with the outcome in patients with AF [4]. The degree of LA fibrosis can be evaluated by delayed enhancement CMR (DE-CMR), however, the high cost, complex operation and time consumption of CMR limit its wide use in clinical practice [34]. At present, echocardiography is still the most practical and convenient imaging tool. The degree of LA fibrosis can be noninvasively evaluated by integrated backscatter (IBS), which can characterize tissue structure based on tissue ultrasound reflectivity [15]. Previous studies have confirmed the excellent relation between IBS and the extent of collagen deposition [35]. In recent years, myocardial strain and strain rate analysis have become a new noninvasive method for indirect evaluation of LA fibrosis [36]. Several studies have demonstrated that the extent of atrial fibrosis is negatively correlated with LA strain and strain rate on DE-CMR [36,37]. Therefore, LA strain analysis plays a significant role in assessing LA fibrosis in patients with AF. Peak LA longitudinal strain (PALS) is negatively associated with the extent of LA fibrosis and is a critical indicator of the recurrence of AF after successful ablation [38]. It is usually measured at the end of LA filling and precisely predicts LV filling pressures [39], consequently, PALS can provide important significance in patients with AF [40].

### 2.2. LA Function Assessment in AF

Under normal physiological conditions, the primary function of LA includes filling and emptying. LA function can be further divided into three aspects. LA respectively serves as a reservoir for pulmonary venous flow during ventricular systole, a conduit for pulmonary venous flow during early ventricular diastole, and a booster pump that increases ventricular filling during late ventricular diastole [41]. The booster pump function is the most important mechanical function of LA, which significantly contributes to approximately 20% to 30% of the left ventricular (LV) stroke volume at the end of LV diastole [27]. LA reservoir function is affected by atrial compliance, LV systolic function and LA contraction and relaxation. While LA conduit function is influenced by atrial compliance during ventricular diastole, it is closely associated with preload and LV relaxation and stiffness. LA booster pump function largely depends on the intrinsic LA contractility, but is also influenced by venous return, LV end-diastolic pressures, and LV systolic reserve [20,42,43]. 

LA functional remodeling is significantly related to LA structural remodeling, and the vicious circle of “AF-induced AF” can be caused by structural remodeling and electrical remodeling of the LA [44]. In these circumstances, both the frequency and duration of AF increase, which are the main reasons for AF maintenance and the conversion to persistent AF. LA booster pump function and reservoir function are usually damaged due to LA structural remodeling in patients with AF, which are manifested as LA dysfunction. Additionally, recent research has demonstrated that LA dysfunction may precede LA structure changes [45,46], and therefore, the evaluation of LA function can provide more prognostic value [47,48,49]. In conclusion, LA function evaluation also plays a crucial role in AF.

## 3. Echocardiographic Assessment of LA Structure and Function in AF

### 3.1. Real-Time Three-Dimensional Echocardiography

**Figure 1:**
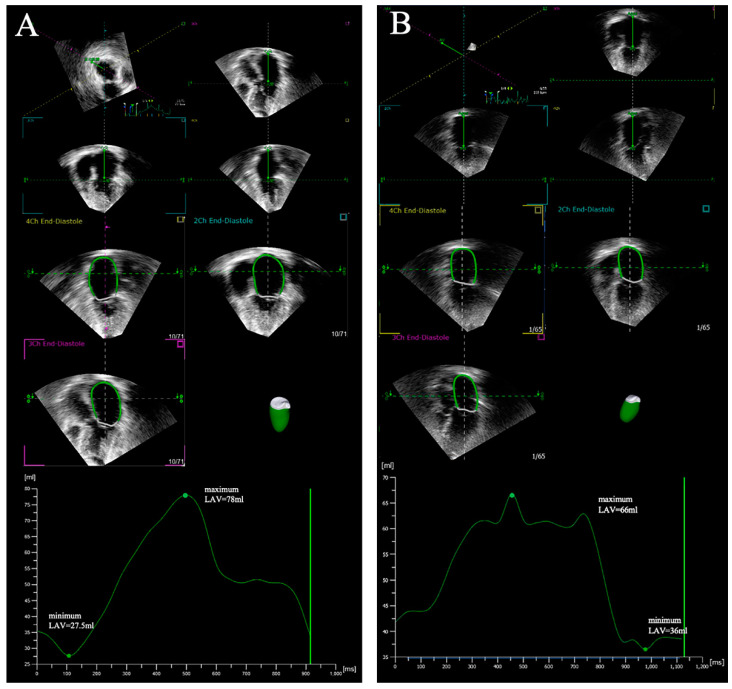
Representative measurements of LAV by three-dimensional echocardiography. Standard apical four-chamber view images of 20 consecutive cardiac cycles were stored, and LAV was measured in one of the cardiac cycles. (**A**) Reference points setting, LA endocardial border tracking and acquisition of 3D LAV curve in a subject with sinus rhythm; (**B**) Reference points setting, LA endocardial border tracking and acquisition of 3D LAV curve in a subject with AF.

LAV is typically evaluated by standard 2D echocardiography. However, traditional 2D echocardiography is usually limited by its poor image quality, higher intra-observer and inter-observer variability, 2D image plane and geometric assumptions, which can underestimate the true LAV. In recent years, the advent of RT-3DE has improved the accuracy and reproducibility of LV volume assessment. Three-dimensional echocardiography can better define the complex anatomy of LA in tri-plane with less geometric assumptions, and it can make up for the error caused by shortening of biplanar volume measurement in 2D plane. Additionally, LAV assessment by 3DE is more closely associated with CMR, the gold standard of LAV assessment [15,20,50,51,52,53,54]. A major shortcoming of 3DE is the comparatively low frame rate for 3D image acquisition and the low spatial resolution, though LAV by 3DE has demonstrated prognostic value [15]. RT-3DE can also assess LA dysfunction by LAVi, maximum LAV and minimum LAV (Figure 1), which can provide independent and incremental prognostic value compared with those above parameters evaluated by 2D traditional echocardiography [55,56,57].

### 3.2. Speckle Tracking Echocardiography

Myocardial strain and strain rate are quantifications of the magnitude and rate of myocardial deformation. LA strain and strain rate can be evaluated by tissue doppler imaging (TDI) and STE. STE can compensate for the limitations of TDI, which including angle-dependence and susceptible to reverberations, sidelobes and drop out artifacts [58,59,60,61]. In recent years, LA strain and strain rate derived from STE have been seen as the gold standard for LA function assessment [62]. LA strain is presented in the form of a time–strain curve and is divided into three phases. The task force of European Society of Cardiology recommends using the QRS complex as the zero baseline for patients with AF, and the ventricular end-diastole as the zero reference. LA myocardial deformation is usually evaluated as global longitudinal strain, which is defined as the strain in the direction tangential to the endocardial atrial border in the apical four-chamber view. Peak LA global longitudinal strain during reservoir phase (LASr) is assessed as the difference of the strain value at mitral valve opening minus ventricular end-diastole. The peak LA global longitudinal strain during conduit phase (LAScd) is evaluated as the difference of the strain value at the onset of atrial contraction minus mitral valve opening. The peak LA global longitudinal strain during contraction phase (LASct) is estimated as the difference of the strain value at the ventricular end-diastole minus onset of atrial contraction. Since the atrial wall lengthens during the reservoir phase, LASr is usually characterized by a positive value. Due to the shortening of the LA wall during the other two phases, LAScd and LASct are usually considered as negative values. In addition, LAScd has the same value as LASr in patients with AF, however the sign is negative [63,64]. Under the atrial cycle, the first negative peak strain of the LA strain curve represents the contraction function of LA (LASct), the positive peak strain corresponds to the conduit function (LAScd), and the sum of the two peaks represents the reservoir function (LASr). PALS also be used to represent LA reservoir function in other studies, and stands for the same significance as LASr. In the same way, peak LA contraction strain (PACS) represents the LA contraction function, the same as LASct, and the difference between PALS and PACS reflects LA conduit function. 

LA function can also be evaluated by volumetric function parameters derived from STE. Total LA emptying fraction (LAEF) indicates the LA reservoir function and can be further divided into passive LAEF and active LAEF, representing conduit function and pump function, respectively [65]. The full name and calculations of LA functional parameters are depicted in Table 1.

#### 3.2.1. Two-Dimensional Speckle Tracking Echocardiography

**Figure 2:**
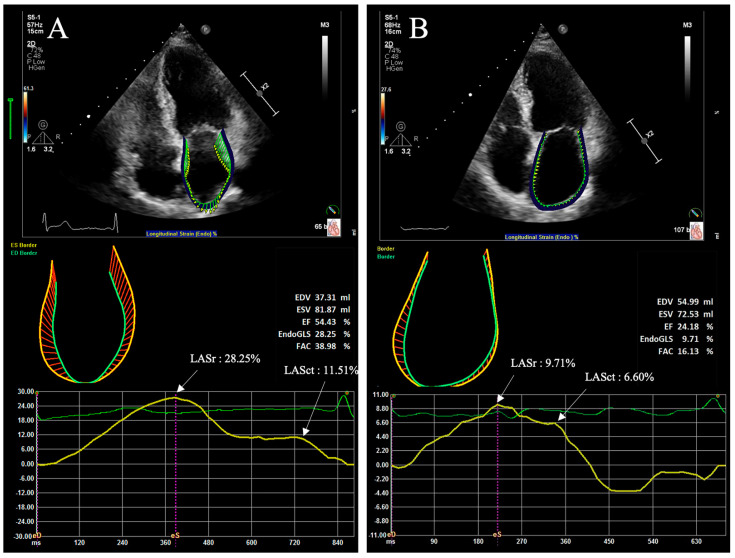
Representative measurements of LA strain by two-dimensional echocardiography. Standard apical four-chamber view images of 20 consecutive cardiac cycles were stored, and LA strain was measured in one of the cardiac cycles. (**A**) LASr and LASct in a subject with sinus rhythm; (**B**) LASr and LASct in a subject with AF.

At present, 2D-STE is the most commonly used technique in evaluating myocardial strain and strain rate [40]. It is a developing ultrasonic technique, and myocardial deformation can be evaluated through acoustic backscatter (speckles) generated by the reflected ultrasound beam. It is preferable to obtain the region of interest (ROI) from a non-foreshortened apical four-chamber or two-chamber view, and the complete ROI of LA is defined as the endocardial border and the epicardial border. The endocardial border of LA usually starts the tracing at the endocardial border of the mitral annulus in the apical four-chamber, and traces the LA endocardial border, extrapolating across the pulmonary veins, and/or LA appendage orifices, up to the opposite mitral annulus side [64]. 2D LASr and LASct in subjects with sinus rhythm and AF are shown in Figure 2. Strain analysis by 2D-STE is a method of myocardial deformation in 2D plane, and that may lead to the out-of-plane motion of speckles. In addition, LA strain assessed by 2D-STE mainly reflects the motion of longitudinal myocardial fibers, which cannot comprehensively describe the complete myocardial function and may overlook LA dysfunction.

#### 3.2.2. Three-Dimensional Speckle Tracking Echocardiography

Three-dimensional STE (3D-STE) is a new technique that combines 3D echocardiography and speckle tracking imaging. It can overcome the shortcomings of 2D-plane tracking and track the motion of myocardium more comprehensively, objectively and accurately in 3D space. Three-dimensional STE can compensate for the limitation of 2D-STE that merely reflects the motion of longitudinal myocardial fibers, and evaluates not only longitudinal strain but also circumferential and area strain [66]. Therefore, 3D-STE can more precisely assess the LA function with excellent reproducibility [67]. However, 3D-STE is largely dependent on image quality and temporal resolution [32,68]. These aspects are the reasons that LA strain by 3D-STE is a better parameter but with lower clinical application rate. 

## 4. LA Structure and Function to Predict the Occurrence of AF

Early prediction of AF in patients with sinus rhythm is of significance to prevent thromboembolism, the major complication and the first clinical manifestation of AF, and other adverse outcomes. LAV has been considered as a significant indicator of the occurrence of AF. Bruun Pedersen et al. [69] assessed the LAVi and maximum and minimum LAV by 2D and 3D echocardiography in 110 patients with transient ischemic attack (TIA) and without the history of AF or stroke. Fourteen patients developed AF during a mean follow-up of 2.2 years. They demonstrated that LAVi derived from both 2D and 3D images was larger in patients with AF, and those patients also had larger maximum and minimum LAV on both 2D and 3D echocardiography. However, only 3D LAVi, not 2D LAVi, was statistically significant in relation to the occurrence of AF. 

The morbidity of AF in patients with Chagas disease is twice that of patients without Chagas disease, and in addition, AF is associated with the incremental mortality of Chagas disease [70,71]. Thus, the predictors of AF in patients with this disease play an important role in early prediction of the outcome of Chagas disease. Saraiva et al. [54] evaluated 392 adult patients with chronic Chagas disease using 3D echocardiography and 2D-STE. They found that 3D total and passive LAEF and 2D LASct had independent predictive value of the occurrence of AF. This study suggested that LA function parameters could provide superior prognostic value to predict new-onset AF in patients with Chagas disease compared with LAV parameters. In addition, this study demonstrated that the LA reservoir function is the best component of LA function that is associated with the occurrence of AF. However, 2D LASct representing LA contractile function was the only 2D LA strain parameter with incremental prognostic value. The above conflicting results may be due to the limitations of a biplane analysis of 2D-STE, far field, LA thin walls, pulmonary veins and LA appendage orifices of LA strain analysis. Therefore, it is necessary to apply 3D-STE to quantify LA function in clinical practice.

Hirose et al. [72] evaluated LAEF, LA strain rate and LAV by STE in 580 consecutive adults without a history of atrial arrhythmias. They demonstrated that, compared to subjects with non-AF at baseline, those without new-onset AF had higher active LAEF and higher LA strain rate during LA systole, but lower maximum LAVi. In addition, only reduced active LAEF was independently associated with new-onset AF in multivariate logistic regression analysis, therefore, active LAEF evaluated by STE was an independent predictor of the risk of new-onset AF. This study demonstrated that LA function parameters could provide superior prognostic value over LA size. 

LA strain can predict the new onset of AF not only in the general population, but also in patients with heart failure (HF). Park et al. [73] researched PALS in 2461 acute HF patients with sinus rhythm, and confirmed that patients with reduced PALS had higher new-onset AF compared to subjects with relatively higher PALS during a 5-year follow-up. PALS could be seen as a significant parameter to predict the risk of new-onset AF in patients with acute HF and sinus rhythm, which could help clinicians to timely adjust treatment decisions. Jasic-Szpak et al. [74] researched PALS, PACS by 2D-STE and LAVi by 2D traditional echocardiography in 170 patients with symptomatic HF with preserved ejection fraction (HFpEF), without baseline AF. They found that PACS, PALS, and LAVi could provide the best predictive value for AF. They demonstrated that predictive value of PACS and PALS was independent from clinical data, LAVi and other conventional LA function parameters, and in addition, PALS and PACS could offer incremental predictive value. Therefore, involving PALS and PACS in the diagnostic algorithm was able to detect the HFpEF patients with high risk of AF, contributing to guiding clinical management. 

A single center cross-sectional study [65] showed that LA enlargement, LA reservoir and pump function, but not conduit function, were independently related to AF. The parameters that derived from 3D-STE, particularly minimum LAV, total LAEF, active LAEF, LASrc and LASctc, were able to distinguish paroxysmal AF subjects from patients without AF. This study demonstrated the relationship between LA strain and LA volumetric function parameters for the first time. They confirmed that the peak LA global circumferential strain during reservoir phase (LASrc), peak LA global circumferential strain during conduit phase (LAScdc) and peak LA global circumferential strain during contraction phase (LASctc) are more significantly associated with total LAEF, passive LAEF and active LAEF, respectively, over LASr, LAScd and LASct. In addition, that may be attributed to the more significant impacts of circumferential myocardial deformation of LA on LA global hemodynamic function. Thus, they assumed that LA circumferential strain might offer greater value. They also hold the opinion that 3D-STE can be used as an accurate tool to determine patients with paroxysmal AF even before structural changes, and therefore it can guide the determination of treatment strategy in daily clinical practice. The researchers reckoned that LASrc, the parameter of LA reservoir function, was of the most outstanding clinical value, because LA pump function of patients with AF disappeared and LA conduit function was less influenced by AF.

Hypertension (HT) is considered the most common risk factor for paroxysmal AF. Furukawa et al. [75] investigated the relationship between LA function and the occurrence of AF by 3D-STE in 44 hypertensive patients with paroxysmal AF, 50 hypertensive patients without paroxysmal AF, and 50 healthy controls without HT or paroxysmal AF. They demonstrated that peak LA global strain and LAEF in patients with paroxysmal AF were obviously lower compared to those without paroxysmal AF and controls. Maximum LAVi and peak LA global strain were independent predictors of paroxysmal AF in patients with HT by multivariate analysis. They confirmed that LA dysfunction is related to the occurrence of AF in patients with HT by using 3D-STE.

Paroxysmal AF is seen as the most common reason of severe stroke and one of the most widespread covert reasons of cryptogenic stroke [76]. The more time spent in paroxysmal AF each day is associated with higher risk of thromboembolism [77]. Pagola et al. [78] demonstrated that PALS and N-terminal pro b-type natriuretic peptide (NT-proBNP) as LA dysfunction indicators could provide significant predictive value for the occurrence of paroxysmal AF with high risk of embolization (HpAF) despite LA size and the age of patients. In addition, the combination of PALS with NT-proBNP could independently predict the occurrence of HpAF with increased predictive power, which was superior to traditional surrogates as age or LAVi. 

## 5. LA Structure and Function to Predict the Recurrence of AF after Catheter Ablation

The critical management of AF is to identify and improve predisposing conditions, restore the normal heart rate and sinus rhythm, and predict and reduce the risk of systemic thromboembolism and stroke. Catheter ablation (CA) is a well-established treatment for restoring and maintaining sinus rhythm in patients with AF [79], and pulmonary vein isolation by radiofrequency or cryoablation is the most common ablation technique [12,80,81]. The Scale for the Assessment and Rating of Ataxia (SARA) research has confirmed that CA was obviously more effective than antiarrhythmic drug treatment in maintaining sinus rhythm for patients with persistent AF, and it decreased the recurrence of 24 hours of sustained episodes of AF by 47.4% [82]. Guidelines increasingly support the use of CA in AF as an early therapeutic approach to symptom control, particularly for symptomatic drug-refractory AF [83]. Whereas LA fibrosis can be aggravated by the energy of CA, the LA structural remodeling and dysfunction can be exacerbated by the scar after ablation. Therefore, after successful ablation, in approximately one-third of patients AF may recur. In addition, AF cannot be eliminated in around 10% of AF patients [84,85,86]. In sum, the determination of parameters of LA remodeling for predicting patients with high risk of recurrence after CA plays a significant role in assisting clinicians in making more specific therapeutic strategy. LA size can determine the risk of recurrence of AF in patients after CA [87]. Additionally, LA strain evaluated by 2D-STE has been demonstrated as an independent predictor of AF recurrence after CA [88,89,90], however, 2D-STE is limited by its dependency on image quality and the out-of-plane motion of some speckles, which may overestimate LA function [68].

Montserrat et al. [91] found that LAV evaluated by 3D echocardiography was a superior predictor of the outcome after first radiofrequency catheter ablation (RFCA) when compared with 2D LAV. They also demonstrated that only the higher LA expansion index (LAEi) was related to non-recurrence of AF after the first RFCA in the multivariate analysis. Additionally, 3D maximum LAV was significantly associated with the non-recurrence of AF in the younger patients than aged 54 or younger after a second RFCA. All in all, this research confirmed that 3D LA function parameter was superior to 3D LA size in predicting the non-recurrence of CA. They recommended the combination of LA function parameters evaluated by 3D echocardiography and that clinical characteristics including age and hypertension were able to improve the predictive ability of the outcome of RFCA.

Yang et al. [92] established a predictive model by using LA reservoir function parameters that measured by RT-3DE, LAEi and diastolic ejection index (DEI), and the blood B-type natriuretic peptide (BNP) level before circular pulmonary vein ablation (CPVA) in 215 patients with early persistent AF after CPVA. LA appendage peak emptying velocity (LAAV) was also evaluated by pulse Doppler imaging in all patients. They found that patients with AF recurrence displayed higher minimum LAVi, DEI and BNP, however, patients with sinus rhythm had higher LAEi and LAAV. The above four parameters, BNP, LAAV, minimum LAVi and DEI, were the optimal predicators after single-factor ROC curve analysis, and a predictive model for recurrence was constructed by DEI, BNP and LAAV after multivariate logistic regression analysis. They demonstrated that the combined predictive model was superior to the best single-factor indicator (BNP) in predicting AF recurrence after CPVA with significantly excellent precision, efficiency and specificity.

Mochizuki et al. [66] estimated LA strain by 2D-STE and 3D-STE in forty-two paroxysmal AF patients undergoing first-time CA. They demonstrated that 3D global peak LA area strain during systole (3D-GASs) was independently associated with AF recurrence and it was demonstrated to be a better predictor of AF recurrence after CA compared with LA strains evaluated by 2D-STE or other clinical predictors, such as LAV, age and so on. This study also explained that it is possible that the single longitudinal myocardial evaluation of 2D-STE and the differences of clinical characteristics (age, gender distribution, LAV and the values of 2D-GLSs) between this study and previous research may be the reasons that 2D LA strain is inferior to 3D LA strain for predicting AF recurrence after CA. In short, this research proved that LA strain derived from 3D-STE is of better prognostic value in predicting the recurrence after CA for patients with paroxysmal AF compared with 2D LA strain parameters and other relevant indicators.

## 6. LA Structure and Function to Predict the Risk of Other Cardiovascular Diseases in AF

### 6.1. Ischemic Stroke

LA enlargement is common in patients with AF and indicates the presence of adverse LA structure remodeling, which is related to electrical instability and easily contributes to thromboembolism formation [93]. AF can increase the risk of ischemic stroke by four- to five-fold by contributing to a prothrombotic status and abnormal endothelial function [2,94]. Thus, early recognition of AF patients at high-risk of stroke plays an imperative role in guiding antithrombotic treatment. However, previous risk stratification schemes were mainly based on baseline demographics and clinical illnesses and ignored the effect of abnormal LA structure and function [95,96]. The increased LAV and damaged function are associated with a relatively higher clinical risk of thromboembolic stroke events. Currently, 2D-STE is emerging as one of the commonly used imaging tools for risk stratification of stroke in patients with AF [17,97]. PALS is the most frequently used indicator for increased risk of ischemic stroke among all LA deformation parameters [98,99], which is increasingly recognized in clinical practice. 

Though 2D-STE is a useful tool in evaluating LA function in AF, it is limited by an irregular heart cycle in patients with AF to a great extent. Liao et al. [100] studied the predictive value of PALS by 2D-STE and the repeatability and feasibility of it by three-beat average method compared with 10-s average, and index-beat measurement among 1457 AF participants. This study confirmed that PALS and LA longitudinal strain rate (LASR) by averaged three-beat method during AF were significantly correlated with other measurements with excellent feasibility and repeatability. They also demonstrated that PALS, LA longitudinal systolic strain rate (LASRs) at the reservoir phase and the absolute value of LA longitudinal early diastolic SR (LASRe) at the conduit phase were associated with the decreased risk of stroke adjusted for clinical covariates or CHA2DS2-VASc score and traditional echocardiography parameters. However, only PALS was still independently associated with ischemic stroke after further adjusting LV global longitudinal strain (LVGLS) and LAVi. Therefore, the employment of PALS was able to offer incremental predictive value of ischemic stroke risk classification in patients with low and high CHA2DS2-VASc score. 

Liao et al. [101] studied the predictive ability of the decreased PALS in 1364 patients with AF. They found that PALS was significantly impaired in patients developing stroke compared with those without stroke. In addition, all patients were classified into five groups according to the standard score of PALS (ZLA): Z0, Z − 1, Z − 2, Z − 3, and Z − 4. During the mean follow-up period of 3.1 ± 1.6 years, the morbidity of patients with Z − 2 to Z − 4 was gradually increased. When the Z − 2 group was defined as a reference, the Z − 3 and Z − 4 groups demonstrated a higher risk of ischemic stroke after adjusting clinical and echocardiographic variables. This study demonstrated that the decreased PALS could be presented in a stratified way, and the classification of PALS was related to the risk of ischemic stroke, which was independent of the baseline covariates, CHA2DS2-VASc score, and traditional echocardiographic parameters.

### 6.2. Heart Failure or Cardiovascular Death 

AF can contribute to LA structural and functional remodeling, which is also correlated with the increased risk for HF and cardiovascular death in addition to stroke [102]. Therefore, it is also crucial to determine AF patients with high risk of HF or cardiovascular death. Wen et al. [103] found that higher LAVs (maximum LAVi and minimum LAVi) and lower LA reservoir function (LAEF and LAEi) at three months after CA were strongly associated with higher risk for HF and cardiovascular death during a median of 7.5 years of follow-up, which was independent of age, atrial tachyarrhythmia recurrence, and clinical and conventional echocardiographic parameters of LV structure and function. In addition, increased minimum LAVi could help determine the patients with high risk of adverse outcome in those with normal LA size. This study also demonstrated that minimum LAVi was the best predictor that related to HF or cardiovascular death compared with maximum LAVi, LAEF and LAEi.

A prospective echocardiographic study [104] demonstrated that increased LAVi and decreased LAEF and LAEi were related to the higher level of risk for HF and cardiovascular death alone in the unadjusted analysis and only impaired LA function indicators, LAEF and LAEi, were significantly associated with HF and cardiovascular death after adjusting clinical and echocardiographic parameters. This study also found that around half of patients with normal LA structure had LA dysfunction, and that was consistent with the results of previous studies that LA dysfunction may occur prior to LA structural abnormality. LA dysfunction was able to increase the risk of HF and cardiovascular death in spite of normal LAV, indicating that LA function parameters could provide incremental prognostic value compared with LAV.

The temporal changes of LA structure and function are of important significance in patients with HF and AF, which may predict the treatment response and prognosis. Many previous studies have researched the prognostic value of LA structure and function parameters in patients with HF and with AF. Mathias et al. [105] studied the response to cardiac resynchronization therapy with a defibrillator (CRT-D) in 533 patients with CRT-D and left bundle branch block (LBBB). The changes of both LAV and left ventricular end-systolic volume (LVESV) were above median changes in patients with complete left-sided reverse remodeling, and only LAV or LVESV changed above the median change in patients with discordant reverse remodeling. They demonstrated that the risk of HF and death, HF alone and death alone in patients with complete left-sided reverse remodeling was significantly lower compared with patients with discordant or lesser reverse remodeling during long-term follow-up. 

Park et al. [106] demonstrated that PALS was a significant predictor of death and HF hospitalization in patients with acute HF and AF. However, PALS was not associated with death and HF hospitalization in patients with AF when they divided patients according to the presence of AF. PALS could predict the adverse outcome when patients were divided according to the type of HF, HF with reduced ejection fraction (HFrEF), HF with mid-range ejection fraction (HFmrEF), or HFpEF, but the prognostic power of PALS was same among the above three subgroups. 

Moon et al. [107] evaluated the time trajectories of the left ventricular global longitudinal strain (LVGLS) and LASr in 409 patients with HFrEF prescribed sacubitril/valsartan. They demonstrated that both the LVGLS and LASr improved over time during follow-up, and the prominent improvements in LAGLS and LASr occurred within six months of sacubitril/valsartan treatment. Larger improvements were associated with death and HF hospitalization, and the improvement in the LVGLS to ≥ 13% and LARS to ≥12.5% could better predict the lower risk of cardiovascular death and HF hospitalization compared with other traditional echocardiographic parameters. This study confirmed that the improvement of LVGLS and LASr, defined as complete left heart reverse remodeling, could be seen as a significant predictor to reflect the treatment response and prognosis.

## 7. Conclusions

LA enlargement and dysfunction can predict the occurrence of AF in patients with sinus rhythm; it was also significantly associated with the adverse outcome of AF. Currently, implementation of RT-3DE and STE has provided a novel imaging technique to assess the LA size and function in AF with more accuracy, sensitivity and repeatability. However, there is lack of relevant studies about the role of 3D-STE in predicting stroke, HF and cardiovascular death in AF and more research is essential to be implemented in the future.

## Figures and Tables

**Table 1:** The full name and calculations of LA structural and functional parameters.

Abbreviation	Full Name	Calculations
LAV	Left atrium volume	Maximum LAV and minimum LAV are measured using the modified Simpson biplane method at the end-systolic frame preceding mitral valve opening and the end-diastolic frame preceding mitral valve closure, respectively
LAVi	Left atrium volume index	Maximum LAVi and minimum LAVi are measured using the modified Simpson biplane method at the end-systolic frame preceding mitral valve opening and the end-diastolic frame preceding mitral valve closure, respectively, and indexed to body surface area
LAEi	Left atrium expansion index	(Maximum LAV–Minimum LAV)/Minimum LAV
Total LAEF	Total left atrium emptying fraction	(Maximum LAV–Minimum LAV)/Maximum LAV
Active LAEF	Active left atrium emptying fraction	(LAVpreA–Minimum LAV)/LAVpreA
Positive LAEF	Positive left atrium emptying fraction	(Maximum LAV–LAVpreA)/Maximum LAV
LASr (PALS)	Peak left atrium global longitudinal strain during reservoir phase (peak left atrium longitudinal strain)	The difference of the strain value at mitral valve opening minus ventricular end-diastole (the peak value of longitudinal strain during LV systole)
LASct (PACS)	Peak left atrium global longitudinal strain during contraction phase (peak left atrial contractile strain)	The difference of the strain value at the ventricular end-diastole minus onset of atrial contraction (the value of strain at the onset of P-wave in electrocardiogram)
LAScd	Peak left atrium global longitudinal strain during the conduit phase	The difference of the strain value at the onset of atrial contraction minus mitral valve opening (LASct minus LASr)
LASrc	Peak left atrium global circumferential strain during reservoir phase	The peak value of circumferential strain during LV systole
LASctc	Peak left atrium global circumferential strain during contraction phase	The difference of the strain value at the ventricular end-diastole minus onset of atrial contraction
LAScdc	Peak left atrium global circumferential strain during conduit phase	The difference of the strain value at the onset of atrial contraction minus mitral valve opening (LASctc minus LASrc)
LASR	Left atrium longitudinal strain rate	LASR: LASR ≈ (V2 − V1)/d, where V2 and V1 are instantaneous velocities measured in two regions of interest, and d is the distance between the two regions of interest; LASRs (Left atrium longitudinal systolic strain rate): the peak positive longitudinal strain rate during LV systole in strain rate curve; LASRe (Left atrium longitudinal early diastolic strain rate): the negative strain rate during early diastole in strain rate curve; LASRa (Left atrium longitudinal late diastolic strain rate): the negative strain rate during late diastole in strain rate curve

LAVpreA: presystolic volume of LA at the beginning of P wave on electrocardiogram.

## Data Availability

Not applicable.
